# Immediate Postpartum Intrauterine Contraceptive Device Insertions in Caesarean and Vaginal Deliveries: A Comparative Study of Follow-Up Outcomes

**DOI:** 10.1155/2016/7695847

**Published:** 2016-08-17

**Authors:** Reetu Hooda, Sonika Mann, Smiti Nanda, Anjali Gupta, Hemant More, Jaikrit Bhutani

**Affiliations:** ^1^Department of Obstetrics and Gynaecology, Pt. BD Sharma Post Graduate Institute of Medical Sciences, Rohtak, Haryana 124001, India; ^2^Haryana Civil Medical Services, University of Health Sciences, Rohtak, Haryana 124001, India; ^3^Pt. BD Sharma Post Graduate Institute of Medical Sciences, Rohtak, Haryana 124001, India

## Abstract

*Background*. Immediate postpartum intrauterine contraceptive device (IPPIUCD) is a lucrative postpartum family planning method which provides effective reversible contraception to women in the delivery setting. Our aim was to study the clinical outcomes of IPPIUCD insertions and compare them as a factor of route of insertion (vaginal versus caesarean).* Methods*. This is a retrospective analytical study done in a tertiary care teaching institute. A Cohort of 593 vaginal and caesarean deliveries with IPPIUCD insertions, over a two-year period, was studied and compared for follow-up results. Outcome measures were safety (perforation, irregular bleeding, unusual vaginal discharge, and infection), efficacy (pregnancy, expulsions, and discontinuations), and incidence of undescended IUCD strings. Descriptives were calculated for various outcomes and chi square tests were used for comparison in between categorical variables.* Results*. Overall complication rates were low. No case of perforation or pregnancy was reported. Spontaneous expulsions were present in 5.3% cases and were significantly higher in vaginal insertions (*p* = 0.042). The incidence of undescended strings was high (38%), with highly significant difference between both groups (*p* = 0.000).* Conclusion*. IPPIUCD is a strong weapon in the family planning armoury and should be encouraged in both vaginal and caesarean deliveries. Early follow-up should be encouraged to detect expulsions and tackle common problems.

## 1. Introduction

Most women do not desire a pregnancy immediately after a delivery but are unclear about contraceptive usage in postpartum period. This results in unplanned and undesired pregnancies, which in turn increases induced abortion rates and consequently maternal morbidity and mortality. In a recent study of postpartum unintended pregnancies 86% resulted from nonuse of contraception and 88% ended in induced abortions [1]. Continuation of these pregnancies is also associated with greater maternal complications and adverse perinatal outcomes. In India, 65% women in the first year postpartum have an unmet need for family planning [2]. Hence, providing contraception in this sensitive period is important.

In India, as in many other countries, postpartum family planning is usually initiated after 6 weeks postpartum. Early resumption of sexual activity coupled with early and unpredictable ovulation leads to many unwanted pregnancies in the first year postpartum. Moreover, in developing countries particularly, women who once go back home after delivery do not return for even a routine postpartum check-up, leave aside contraception. This is may be due to lack of education and awareness, social pressure, and nonaccess to facilities nearby.

Thus, immediate postpartum family planning services need to be emphasized wherein the woman leaves the hospital with an effective contraception in place. Increase in hospital deliveries provides an excellent opportunity to sensitize women and provide effective contraception along with delivery services. An intrauterine contraceptive device (IUCD) has several advantages for use in postpartum period as it is an effective, long term reversible contraception, is coitus independent, and does not interfere with breast feeding.

Cochrane reviews provide evidence of safety and feasibility of postpartum IUCD (PPIUCD) insertions in various settings [3, 4]. However, studies have reported high expulsion rates (10.4–16.4%) [5–8]. Most of the studies published were carried out more than a decade ago. Since then various advancements have been tried to decrease expulsion rates and improve PPIUCD acceptance. PPIUCD insertions via different routes (vaginal or caesarean) may have different outcomes at follow-up. There is minimal research comparing results between vaginal and caesarean insertions. Moreover, new understanding of this postpartum contraception necessitates examination of advantages and disadvantages of PPIUCD from a new perspective. This stimulated us to analyze the PPIUCD insertions at our institute.

## 2. Material and Methods

Immediate postpartum IUCD (IPPIUCD) insertions at Pt. B.D. Sharma Post Graduate Institute of Medical Sciences were studied. Follow-up clinic visits of women who reported for examination after 6 weeks of IPPIUCD insertion at our institute were analyzed.

Inclusion criteria for IPPIUCD insertions were women delivering either vaginally or by caesarean section, had received counselling for postpartum contraception, and consented to IPPIUCD insertions. Counselling was done during antenatal visits or during early labour and a written informed consent was taken prior to insertions. Criteria used for exclusion were haemoglobin less than 8 gm%, rupture of membranes more than 18 hours, postpartum haemorrhage, coagulation disorders, fever, or clinical symptoms of infection during labour. The IUCD used was CuT-380 A, which was available free of cost in the Government Program. This was placed in uterine fundus with the help of long and curved forceps without lock (Kelly's Placental Forceps) for vaginal insertions, within 10 minutes of removal of placenta. During caesarean section ring forceps were used to place the IUCD in fundus of uterus through the lower segment incision which was closed subsequently as routine. The IUCD strings were not trimmed in both types of insertions and left in uterine cavity. Active management of third stage of labour was performed as routine. All IPPIUCD insertions were done by doctors who had been trained for this purpose. Postinsertion counselling was done and women were advised to follow-up for examination at our centre after 6 weeks.

At the follow-up visit, the women were asked for any symptoms of unusual vaginal discharge, irregular bleeding per vaginum, and any expulsions noticed. Pelvic examination was performed to examine the descent of IUCD strings into vagina and to check signs of infection and bleeding. Descended strings were trimmed approximately 2 cm beyond external os. If strings were not visible on per speculum examination, an ultrasound was performed to check for expulsions and confirm presence of intrauterine IUCD. If the women requested removal of IUCD for any medical or personal reason, she was counselled and intrauterine device was removed. Women were offered reinsertion of IUCD or alternative methods of contraception in case of expulsions/removals.

Immediate postpartum IUCD service became a Government of India approved program in 2010. Since then IPPIUCD insertions are a part of routine curriculum at this institute. Written informed consent was obtained from all clients of IPPIUCD.

The primary outcome measures were the clinical outcomes in terms of safety (perforation, unusual vaginal discharge, infection, and irregular bleeding), efficacy (pregnancy, expulsions, and discontinuations), and incidence of undescended IUCD strings. These outcomes were compared for vaginal and caesarean IPPIUCD insertions.

Statistical analysis was carried out using Statistical Package for Social Sciences (SPSS) Version 19.0. Descriptives were calculated for various clinical outcomes, and chi square tests were used for comparison in between categorical variables. For all the tests performed, results were considered statistically significant for *p* < 0.05.

## 3. Results

A total of 593 immediate postpartum IUCD insertions were studied. Out of these 346 (58.3%) insertions were intracaesarean and 247 (41.7%) IUCDs were placed after vaginal delivery.

Follow-up clinic visits of IPPIUCD clients recorded were 171 (28.8% of total insertions). Fifty-five percent of the total follow-up visits were of intracaesarean IPPIUCD insertions, but the difference in follow-up visits of vaginal and caesarean IPPIUCDs was not significant (*p* = 0.288).  Table 1 summarizes the outcomes at follow-up visits of all PPIUCD insertions. There was no case of uterine perforation or any unplanned pregnancy.

Symptoms of unusual vaginal discharge were reported by 12.3% women at follow-up and this complaint was significantly higher after caesarean IUCD insertions (*p* = 0.037) (Table 2). On clinical examination, however, only one case of pelvic inflammatory disease and two cases of bacterial vaginosis were detected. In the remaining 18 cases the “discharge” was normal leucorrhoea.

Change in bleeding pattern, which was mainly increased blood loss (menorrhagia), was observed in 10.5% women. There was no significant statistical difference in rates of infection or irregular bleeding between the two insertion groups (Table 2).

Spontaneous expulsion of IUCD occurred in 9 (5.3%) cases at follow-up. One IUCD which was partially expelled into cervical canal was also included in expulsions. Women who had IUCD inserted after vaginal delivery had significantly higher expulsion rates (9.1%) than intracaesarean IUCDs (2.1%) with *p* = 0.042 (Table 3).

IUCD removal was done on request of the women for medical/personal reasons leading to discontinuation in 7 cases (4.1%).

IUCD strings had not descended into vagina in 38% cases at clinical examination done at follow-up visits (the cases of spontaneous expulsions were excluded). All women with undescended strings underwent ultrasonographic confirmation of intrauterine placement of the device. Half of the intracaesarean insertions (55.1%) presented with undescended strings at follow-up as compared to 22.1% insertions after vaginal delivery. This difference was highly significant statistically (*p* = 0.000).

## 4. Discussion

The revival of postpartum IUCD by Ministry of Health and Family Welfare, Government of India, with technical assistance from Jhpiego in 2010 leads to conscious efforts to provide the benefits of this long term reversible postpartum contraception in the delivery setting of our institute [9].

Women undergoing caesarean section seem to have greater probability of accepting postpartum IUCD possibly due to postcaesarean conception fear. Further, the number of women following up after intracaesarean insertions was also higher than postplacental vaginal insertions, although this difference was not statistically significant. It appears that women undergoing caesarean delivery are more compliant with follow-up visits probably for fear of complications.

Although all the women who underwent immediate postpartum IUCD insertions (vaginal or caesarean) were counselled and advised to come for a follow-up examination at our institute, only a few women actually reported for a follow-up clinic visit. The possible explanation could be that even though a large number of rural women from all over our state and neighbouring districts come to our tertiary centre for purpose of delivery, for follow-up examination they prefer visiting their local health centres due to large distances and transportation problems.

In a recent prospective study of follow-up of PPIUCD from a peripheral health centre of India, scheduled follow-up was observed in 65.2% cases. Around 22% cases had to be contacted telephonically and transportation incentives were provided for coming for follow-up [10]. Shukla et al. reported a follow-up of 78.7% in a prospective longitudinal study [5]. The other reason for the poor follow-up in the present study could be that it is a retrospective one.

Amongst the women studied at follow-up, there was no case of uterine perforation. None of the studies, as per literature search, have reported uterine perforation after PPIUCD insertion.

In women reporting symptoms of unusual vaginal discharge, actual infection was present in only 1.75% cases on clinical examination. It is known that some women report increased vaginal discharge with the IUCD, which is usually normal leucorrhoea and not a sign of infection [11]. Women delivering by caesarean section seem to be more apprehensive regarding symptoms of discharge, having undergone a surgical procedure. A multicentric follow-up study from India reported an overall infection rate of 4.5% among PPIUCD insertions [9]. Welkovic et al. compared infection rates among women with postplacental IUD and women without IUD and found no difference [12]. Some studies have found no incidence of infection after PPIUCD insertion [5, 13, 14].

The symptom of irregular bleeding per vaginum was not influenced by route of insertion. The women mainly complained of excessive bleeding and were treated adequately with Nonsteroidal Anti-Inflammatory Drugs (NSAIDs) and haematinics. Shukla et al. indicated a higher incidence of menorrhagia (27.2%) with use of CuT 200 in postpartum women [5]. Gupta et al. observed bleeding in 4.3% PPIUCD cases using CuT-380-A [14]. Other studies using CuT-380 A have reported IUCD removal due to bleeding/pain as 6% to 8% [10, 13]. Difference in types of IUCD could possibly explain the different rates of bleeding problems.

In the present study, a lesser number of spontaneous IUCD expulsions were observed as compared to other studies. Çelen et al. reported 1-year cumulative expulsion rates of 12.6% and 17.6% in two different studies of PPIUCD insertions [6, 13]. In a recent study by Kittur and Kabadi, using similar technique and timing (within 10 minutes of placental delivery) of PPIUCD (CuT-380 A), as in our study, and also trained providers resulted in similar fewer expulsions (5.23%) as in the present study [10]. Timing of IUCD insertion is an important determinant of expulsions. UN-POPIN report stated that 6-month cumulative expulsion rate was 9% for immediate postplacental insertions (within 10 minutes) compared with 37% for insertions between 24 and 48 hours after delivery [15].

The expulsions were significantly higher in postplacental IUCD insertions after vaginal deliveries as compared to caesarean insertions. This difference was also observed in a recent systematic review of PPIUCD insertions [16]. Gupta et al. also reported lower expulsions after intracaesarean insertions [14]. Letti Müller et al. studied expulsion rates of immediate postplacental CuT-380 A insertion by transvaginal sonography and found statistically significant higher expulsions in vaginal insertions than caesarean insertions [17].

In the present study, even if we combine the discontinuations (removal of IUCD for different medical or personal reasons) and spontaneous expulsions we still have a commendable IUCD continuation rate of 90.6%. In the absence of IPPIUCD insertions, these women would have left the hospital premises without effective postpartum contraception. Similar rates of removal of PPIUCD have been reported in recent studies, ranging 3–8% [6, 9, 10, 13].

One of the main observations at follow-up was that of undescended IUCD strings. The practice of leaving the full length of IUCD string in uterine cavity during caesarean section and not passing it through the cervix, unlike study by Çelen et al., may have had a role in the significant difference in the incidence of undescended strings in intracaesarean insertions. Our technique might also be the reason for lower expulsion rates as compared to study by Çelen et al. (5.3%) for intracaesarean IUCD insertions at 6 weeks of follow-up [13]. Counselling the women and confirmation of IUCD in uterine cavity by ultrasound are important to reassure the women and encourage them to continue with the device.

## 5. Conclusion

Insertion of IUCD in immediate postpartum period is an effective, safe, and convenient contraceptive intervention in both cesarean and vaginal deliveries. Although there is a relatively higher incidence of expulsions after vaginal IPPIUCD insertions, they should be encouraged considering the advantages that come along. PPIUCD insertions by trained clinicians, principles of fundal placement using long placental forceps, and timing of insertion are instrumental in reducing complications and expulsions. Early follow-up examinations are important to identify spontaneous expulsions and provide alternative contraceptives or IUCD reinsertions.

## Figures and Tables

**Table 1:** Outcomes of PPIUCDS at follow-up visits.

	Frequency (*n* = 171)	Percentage (%)
*Safety*		
(i) Perforation	0	0%
(ii) Unusual vaginal discharge	21	12.3%
(iii) Infection	3	1.75%
(a) Vaginitis	2	1.17%
(b) PID	1	0.58%
(iv) Irregular bleeding	18	10.5%
*Efficacy*		
(i) Pregnancy	0	0%
(ii) Expulsion	9	5.3%
(iii) Discontinuation	7	4.1%
*Undescended IUCD strings*	65	38%

**Table 2:** Assessment of safety.

		Vaginal *n* = 77	Caesarean *n* = 94	Total *n* = 171	*p* value	Odds ratio
Perforation	No	77	94	171	—	—
	Yes	0	0	0		

Unusual vaginal discharge (self-reported)	No	72	78	150	**0.037**	2.621
	Yes	05	16	21		

Infection	No	76	92	168	0.681	1.638
	Yes	01	02	03		

Irregular bleeding per vaginum	No	67	86	153	0.343	0.6553
	Yes	10	08	18		

**Table 3:** Comparison of efficacy.

		Vaginal *n* = 77	Caesarean *n* = 94	Total *n* = 171	*p* value	Odds ratio
Pregnancy	No	77	94	171	—	—
	Yes	0	0	0		

Expulsion	No	70	92	162	**0.042**	4.273
	Yes	07^*∗*^	02	09		

Discontinuation^*∗∗*^ (removal)	No	72	92	164	0.152	3.052
	Yes	05	02	07		

^*∗*^One expulsion was partial expulsion.

^*∗∗*^Removal on patient request.

## Abbreviations

IUCD: Intrauterine contraceptive device
IPPIUCD: Immediate postpartum IUCD
CuT: Copper-T.
